# Supplementary material for: Modelling the transmission dynamics of Campylobacter in Ontario, Canada, assuming house flies, Musca domestica, are a mechanical vector of disease transmission
Source: R Soc Open Sci. 2019 Feb 13;6(2):181394. doi: 10.1098/rsos.181394 (PMC6408420; doi:10.1098/rsos.181394)
Supplement: Table 1A [file rsos181394supp1.docx]

Table 1A: Univariate sensitivity analysis on initial conditions estimated through parameterization to campylobacteriosis incidence reported to Public Health Ontario in 2005.

| **Initial Condition** | **Value** | **Minimum Test Value** | **Maximum Test Value** | **Percent Change from Minimum (%)** | **Percent Change from Maximum (%)** |
| --- | --- | --- | --- | --- | --- |
| S_h_0 | 8 x10^6^ | 1x10^6^ | 1.3 x10^7^ | -57.57 | 6.29 |
| B0 | 2.5 x10^-2^ | 1 x10^-3^ | 1 x10^-1^ | -55.88 | 55.88 |
| S_f_0 / w | 4910 | 100 | 10000 | -3.45 | 3.65 |
