# Supplementary material for: Modelling the transmission dynamics of Campylobacter in Ontario, Canada, assuming house flies, Musca domestica, are a mechanical vector of disease transmission
Source: R Soc Open Sci. 2019 Feb 13;6(2):181394. doi: 10.1098/rsos.181394 (PMC6408420; doi:10.1098/rsos.181394)
Supplement: Table 2A [file rsos181394supp2.docx]

Table 2A: Percentage increase of *Campylobacter* incidence under climate change scenarios that affect the fly population size and amount of fly activity.

| **Scenario** | **Percent Increase in annual incidence (%)** |
| --- | --- |
| **Baseline** | 25.04 cases / 100,000 population |
| **Fly Population Size Increase** |  |
| Medium-low Emissions: |  |
| 2020: 45.7% | 1.90 |
| 2050: 84.3% | 3.77 |
| 2080: 156% | 6.64 |
| High Emissions: |  |
| 2020: 45.7% | 1.90 |
| 2050: 128% | 5.41 |
| 2080: 244% | 10.35 |
| **Fly Activity Increase** |  |
| 25% | 23.43 |
| 50% | 46.87 |
| 75% | 70.30 |
| 100% | 93.74 |
| **Combinations** |  |
| 25 % activity increase |  |
| 45.7% population size increase | 25.81 |
| 84.3% population size increase | 28.15 |
| 128% population size increase | 30.20 |
| 156% population size increase | 31.74 |
| 244% population size increase | 36.37 |
| 50% activity increase |  |
| 45.7% population size increase | 49.72 |
| 84.3% population size increase | 52.53 |
| 128% population size increase | 54.99 |
| 156% population size increase | 56.83 |
| 244% population size increase | 62.39 |
| 75% activity increase |  |
| 45.7% population size increase | 73.63 |
| 84.3% population size increase | 76.90 |
| 128% population size increase | 79.78 |
| 156% population size increase | 81.93 |
| 244% population size increase | 88.41 |
| 100% activity increase |  |
| 45.7% population size increase | 97.53 |
| 84.3% population size increase | 101.28 |
| 128% population size increase | 104.56 |
| 156% population size increase | 107.02 |
| 244% population size increase | 114.43 |
